# Tislelizumab combined with GT chemotherapy for intimal sarcoma of inferior vena cava: A case report

**DOI:** 10.1097/MD.0000000000038056

**Published:** 2024-05-24

**Authors:** Haihong Liao, Yong Fang, Da Li, Yuefen Pan, Zhongfeng Niu, Tianhong Fu, Zhuoxuan Wu, Jin Sheng, Yong Dong, Shuwen Han, Quan Qi, Yulong Liu

**Affiliations:** a Department of Medical Oncology, Huzhou Central Hospital, Huzhou, Zhejiang, China; b Fifth School of Clinical Medicine of Zhejiang Chinese Medical University (Huzhou Central Hospital), Huzhou, Zhejiang, China; c The Affiliated Central Hospital of Huzhou University, Huzhou, Zhejiang, China; d Department of Oncology, Sir Run Run Shaw Hospital, Zhejiang University School of Medicine, Hangzhou, Zhejiang, China; e Department of Oncology, the Second Affiliated Hospital of Soochow University, Suzhou, Jiangsu, China; f State Key Laboratory of Radiation Medicine and Protection, School of Radiation Medicine and Protection, Medical College of Soochow University, Suzhou, Jiangsu, China; g Collaborative Innovation Center of Radiological Medicine of Jiangsu Higher Education Institutions, Suzhou, Jiangsu, China.

**Keywords:** imaging, immunohistochemistry, inferior vena cava, intimal sarcomas, tislelizumab

## Abstract

**Rationale:**

Intimal sarcoma of inferior vena cava (IVC) is a rare soft tissue sarcoma with no typical symptoms and specific imaging features in the early stage, and there is a lack of standardized treatment and methods.

**Patient concerns:**

A 54-year-old female patient presented to Fenghua District People’s Hospital with a post-active cough and hemoptysis and was subsequently referred to our hospital.

**Diagnoses:**

The patient was pathologically diagnosed as intimal sarcoma of IVC complicating multiple intrapulmonary metastases. Chest CT revealed left lung malignant tumor with multiple intrapulmonary metastases; while enhanced upper abdominal CT showed cancer embolus of IVC with extension to right atrium and bilateral renal veins. Besides, hematoxylin and eosin staining suggested intimal sarcoma of veins. Immunohistochemical staining showed positivity for PD-L1, Ki-67, CD31, Desmin and ERG.

**Interventions:**

The patient initially received GT chemotherapy (gemcitabine injection + docetaxel). Then, immunotherapy (tislelizumab) was added based on the results of genetic testing (TP53 gene mutation).

**Outcomes:**

The disease was stabilized after receiving the treatment.

**Lessons:**

Given the lack of characteristic clinical manifestations in patients with intimal sarcoma of IVC, imaging examination combined with immunohistochemical index were helpful for diagnosis of intimal sarcoma of IVC. Furthermore, the combination of tislelizumab and GT chemotherapy was feasible in such patients with positive PD-L1 expression and TP53 mutation.

## 1. Introduction

Intimal sarcoma is non-myogenic sarcoma originating from the tunica intima or related with large blood vessels.^[1]^ It has been reported that intimal sarcoma accounts for only 1% of all sarcomas, which is commonly seen in aorta and pulmonary artery, but are rare in inferior vena cava (IVC).^[2]^ It occurs mostly in older age groups, with an average age at diagnosis of 48 years for pulmonary intimal sarcoma patients and 62 years old for subjects with peripheral large veins or aortic intimal, respectively.^[3]^ So far, the exact diagnosis of intimal sarcoma of IVC remains unclear. It is difficult to distinguish intimal sarcoma from thromboembolism or cancer embolus due to similar clinical symptoms and imaging findings, resulting in delayed diagnosis and treatment.^[4]^

Afzal et al reported on a patient diagnosed with undifferentiated intimal sarcoma who underwent IVC resection from the right atrium to the infrarenal IVC. After reconstruction of the IVC, the patient showed good tolerance to gemcitabine and docetaxel.^[2]^ On this basis, we highlighted a clinical female case of intimal sarcoma of IVC with extension to the right atrium and bilateral renal veins, accompanied by multiple intrapulmonary metastasis, which is the first case reporting the effectiveness of tislelizumab combined with GT chemotherapy (gemcitabine injection + docetaxel) for PD-L1-positive IVC intimal sarcoma complicating TP53 mutation.

## 2. Case presentation

A 54-year-old Chinese woman presented to Fenghua District People Hospital due to cough with hemoptysis once after activity. A chest computed tomography (CT) scan showed space occupying in the left upper lung, suspected to be a malignant tumor. For further diagnosis and treatment, she was admitted to our hospital.

Physical examination revealed pain score of 0 point, a clear state of mind, good spirit, no evidence of yellowish skin and sclera, obvious murmur on bilateral carotid artery or heart enlargement, flaccid nape, trachea in the middle, normal superficial lymph nodes, normal bilateral lung breath sounds, uniform heart rhythm, flat abdomen without tenderness, percussion tenderness over kidney (−) and normal limb muscle strength.

**Figure 1.**
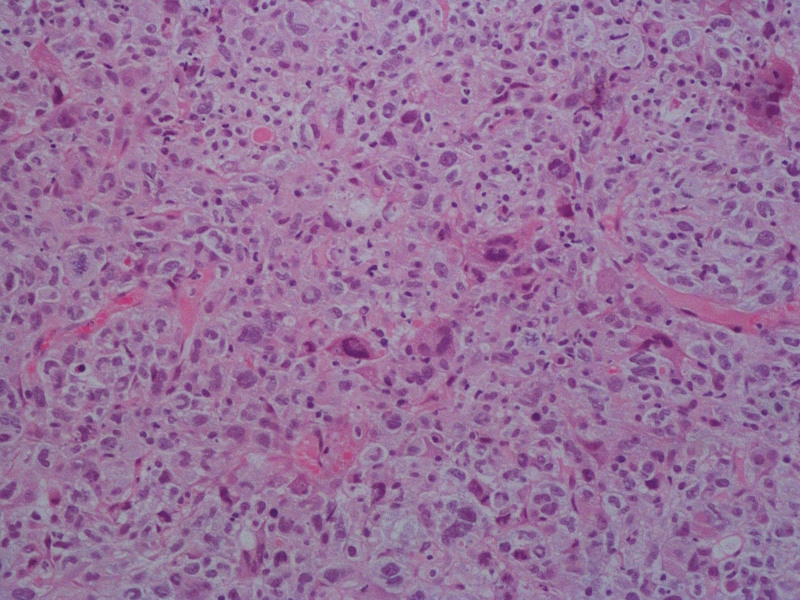
Hematoxylin and eosin staining image. Magnification: ×200.

**Figure 2.**
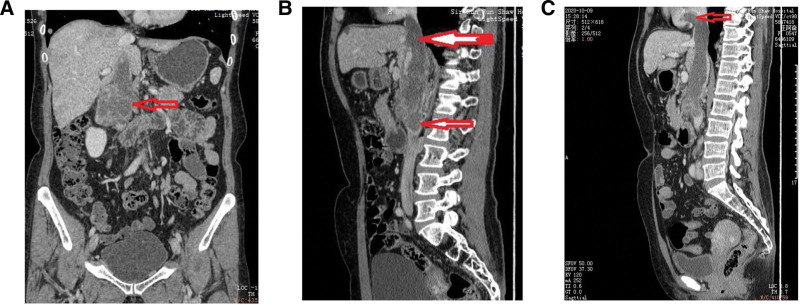
(A) Contrast-enhanced CT coronal view of the abdomen showed the primary lesion of the inferior vena cava. (B–C) Contrast-enhanced CT coronal view of the abdomen showed that the carcinoma thrombin of IVC extended to the right atrium. CT = computed tomography, IVC = inferior vena cava.

The chest CT revealed left lung malignant tumor with multiple intrapulmonary metastases. Under CT guidance, an occupancy biopsy of the lower lung space was performed. The puncture tissue samples were fixed in 10% formalin, followed by paraffin embedding and staining with hematoxylin and eosin. Pathological results showed an intimal sarcoma of veins (Fig. 1). In addition, enhanced upper abdominal CT suggested cancer embolus of IVC with extension to right atrium, bilateral renal veins and lateral branches in the hepato-renal space (Fig. 2A–C).

**Figure 3.**
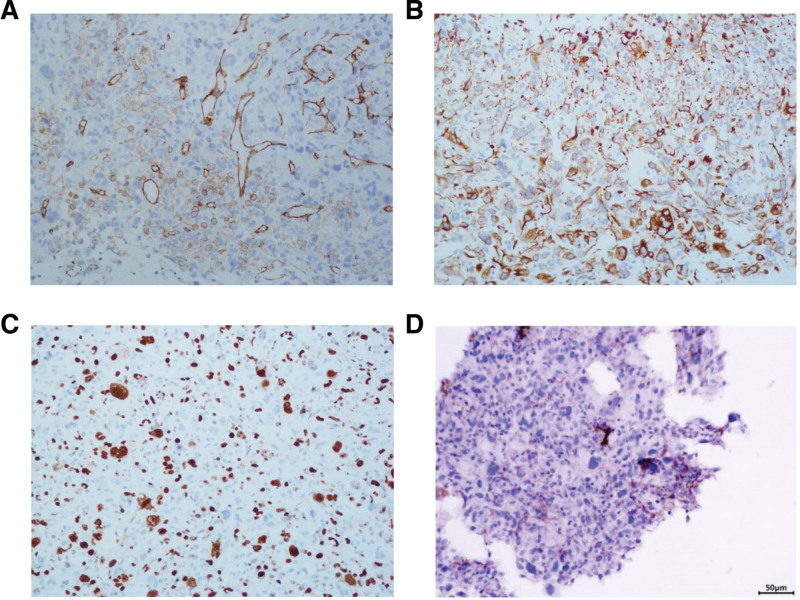
Immunohistochemical staining showed (A) CD31 (+), (B) Desmin (+), (C) Ki-67 (+), and (D) PD-L1 positivity (tumor proportion score = 1%). Magnification: ×200.

Immunohistochemical staining showed positivity for Ki-67 (about 60%+), CD31 (+) and Desmin (+) (Fig. 3A–C), ERG (weak +), TTF-1 (−), NapsinA (−), CK5/6 (−), P40 (−), CK-pan (−), CgA (−), Syn (−), CD56 (−), CD34 (−), S-100 (−), EMA (−), Myogenin (−), MyoD-1 (−), Caldesmon (−), and SMA (−). Similarly, PD-L1 (+) was also confirmed by immunohistochemical staining, which tumor proportion score = 1% (Fig. 3D). Unexpectedly, there was a complete loss of MLH1, MSH2, MSH6, and PMS2 expression. The absence of these mismatch repair proteins may lead to an increased rate of point mutations.

Consequently, the next generation sequencing (NGS) was carried out on the biopsy samples via Illumina Hiseq platform, which covers exon regions of 550 cancer-related genes. Results revealed mutation in exon8 of the TP53 (c.811G > T, p.E271*), with a mutation abundance of 47.6%. The tumor mutation load was 2.0 mutations/Mb, which was lower than the reference value (8.1 mutations/Mb). The results of hyper-progression assessment of immunotherapy clarified DNMT3A (−), EGFR (−), MDM2 (−), MDM4 (−). The patient was therefore diagnosed with IVC endothelial sarcoma.

On August 28,2020, the patient started up to 4 cycles of GT chemotherapy [gemcitabine injection (1.5 g on day 1 and day 8) combined with docetaxel (100 mg on day 1)]. Then, she was administrated with the combination of GT chemotherapy and immunotherapy for 3 cycles based on the results of NGS. Dosage regimen: gemcitabine injection (1.5 g on day 1 and day 8) + docetaxel (100 mg on day 1 and day 8) + tislelizumab (200 mg on day1, q3w). After 2 cycles of combined treatment, the CT reexamination results suggested that the malignant tumor of IVC extended to the proximal ends of the renal veins on both sides, and also extended upward to the right atrium. Compared with the Figure 2, the maximum diameter of the tumor was slightly increased, and some retroperitoneal lymph nodes were enlarged. According to the evaluation criteria of RCISIT1.1, the patient did not reach the state of disease progression.

From January 9 to March 17, 2021, the patient successively underwent ITV-based stereotactic radiotherapy for pulmonary metastases (6MV-X SAD100 DT3000Gy/3f/4d, every other day), GTV-based portal vein tumor plug radiotherapy (6MV-X SAD100 DT3600cGy/12f/16d) and CTV-based portal vein tumor plug radiotherapy (6MV-X SAD100 DT3000cGy/12f/16d). Meanwhile, she also received multiple cycles of gemcitabine (1.5 g on day 1 and day 8) combined with tislelizumab (200 mg on day 1, q3w) from February 3, 2021 to the present, and is in stable condition now. This study was approved by the Ethics Committee of Huzhou Central Hospital (No. 202107005-01), and written informed consent was obtained from the patient.

## 3. Discussion

Primary sarcoma can be classified as vascular wall sarcoma and intimal sarcoma based on vascular tissues. The former is generally derived from the middle layer of the great veins and is relatively easy to diagnose because of its typical morphological features. The latter is a malignant mesenchymal tumor that exhibits fibroblastic or myofibroblastic differentiation.^[5]^ Unlike the vascular wall sarcoma, intimal sarcoma is not easy to classify from their histological pattern and appears as a polyp-like growth into the vascular lumen.^[6]^ The incidence of intimal sarcoma of pulmonary artery has been reported to be nearly twice of the aorta, primarily involving the proximal end of the blood vessel.^[1]^ So far, approximately 200 intimal sarcoma cases have been reported. In a review of studies in the last 10 years, apart from the report by Afzal et al, only 1 article published by Zhang et al in 2019 reported a 66-year-old case who developed high-grade undifferentiated intimal sarcoma that extended from IVC with involvement of nearly whole right atrium, venae iliaca communis and bilateral renal vasculature.^[7]^

Clinically, intimal sarcoma of the IVC may be overlooked initially by both the patient and the clinician, and may even be misdiagnosed as thromboembolism or cancer embolus in the absence of typical symptoms and signs.^[8]^ In the present study, although the patient was in advanced stage of intimal sarcoma, there was no obvious clinical symptoms at the beginning, and hemoptysis occurred only once during routine walking after meals. As the patient was alert, chest plain CT was carried out at the local hospital, results revealed space occupying in the left upper lung, which was considered as pulmonary malignancy with multiple intrapulmonary metastases that complicated with venous cancer thrombus.

Clinically, the diagnosis of IVC intimal sarcoma mainly rely on CT enhanced examination. To our knowledge, thromboembolism is characterized by low-density with no significant enhancement; in the case of cancer thrombus, there may be vascular formation around the embolus, with obvious enhancement of the lesions. However, both are confined to the vascular cavity, showing a filling defect with a normal gap between the embolus and vessel wall.^[2,9]^ For tumors originating in the lumen, the initial site of the tumor may show an aggressive growth in all directions (Fig. 2A), which can be used as a key point of differentiation. In the study, abdominal CT examination of the patient revealed mass filling defects from the right renal vein to the right atrium and bilateral renal veins, locally protruding out of the cavity with a larger layer of 52 × 48 × 177 mm. In addition, there was uneven enhancement of the lesions, advancing forward into the descending duodenum and extending into the renal vein at the lower margin, with a large cross section of the pancreatic head mass and multiple tortuous lateral branches in hepato-renal space. Taken together, the imaging data indicated cancer thrombus.

Ibrahim et al have clarified that tissue sampling and immunohistochemical analysis are gold standard for the diagnosis of intimal sarcoma.^[10]^ We performed biopsy of lung lesions, but due to poor differentiation of the tumor and lack of clear differentiation direction, immunohistochemical indexes related to nerve, smooth muscle and epithelial markers were all negative. Only CD31, ERG, Ki-67, and Desmin were positive, and malignant mesenchymal tumor was considered. After careful discussion among pathology department, imaging department and clinical department, the patient was finally diagnosed as primary intimal sarcoma of IVC.

Due to its rarity, there is no clear consensus concerning on the function of systemic therapy in intimal sarcoma of IVC. Currently, surgery, radiotherapy, targeted therapy and chemotherapy are published treatments for intimal sarcoma,^[11]^ among which surgical resection is the mainstay for short-term remission.^[12]^ However, for newly diagnosed patients with direct infiltration or metastasis to lung parenchyma, the opportunity for surgery has been lost, with a mean survival of only 14 to 18 months. The patient in our case was initially given a combination of gemcitabine and docetaxel rather than surgery after diagnosis, since she presented with multiple intrapulmonary metastasis.

Immune system plays a critical role in the progression of cancer, and proper regulation of the immune system may provide effective therapy options for sarcomas.^[13]^ To find out related targets, we performed NGS and found TP53 mutation (c.811G > T p.E271*). Besides, immunohistochemical staining also revealed positivity for PD-L1. The frequency of PD-L1 expression in sarcomas varies greatly, ranging from 0% to 65%.^[14]^ An integrative analysis of clinical cohort has suggested that NSCLC patients who carry TP53 gene mutation are more susceptible to PD-1 inhibitor therapy.^[15]^ Tislelizumab is a PD-1 receptor inhibitor of humanized IgG4 monoclonal antibody with high specificity and affinity, which binds to the extracellular domain of human PD-1 and blocks the binding of PD-L1 and PD-L2.^[16]^ In a single-arm phase 2 trial conducted by Ye et al, tislelizumab has shown a meaningful clinical benefit in patients with locally advanced or metastatic PD-L1 positive urothelial carcinoma.^[17]^ Based on the genetic results, tislelizumab immunotherapy was added. The patient underwent CT examination after 2 cycles of the combined treatment, and the results showed that the primary lesion was stable and the lung lesion was reduced, indicating that the treatment was effective.

## 4. Conclusion

We report this case to provide support for further understanding of intimal sarcoma in the IVC, especially in cases with vascular filling defect. The imaging results should be carefully reviewed to avoid misdiagnosis and missed diagnosis, and the combination of immunohistochemical indicators is helpful for diagnosis of intimal sarcoma in the IVC. Moreover, combined therapy of tislelizumab and GT chemotherapy contributes to the control of this disease, indicating that NGS can be used to identify immunotherapy targets to customize immunotherapy, thereby improving therapeutic efficacy.

## Author contributions

**Conceptualization:** Haihong Liao, Yong Fang, Da Li.

**Investigation:** Yuefen Pan, Zhongfeng Niu, Tianhong Fu.

**Resources:** Yong Dong, Shuwen Han.

**Supervision:** Quan Qi.

**Validation:** Zhuoxuan Wu, Jin Sheng.

**Writing – original draft:** Haihong Liao.

**Writing – review & editing:** Haihong Liao, Yulong Liu.
